# Frequent Genic Rearrangements in Two Regions of Grass Genomes Identified by Comparative Sequence Analysis

**DOI:** 10.1002/cfg.164

**Published:** 2002-04

**Authors:** Wusirika Ramakrishna, Jianxin Ma, Phillip SanMiguel, John Emberton, Jorge Dubcovsky, Bryan A Shiloff, Zeyu Jiang, Nils Rostoks, Carlos S. Busso, Matthew Ogden, Eric Linton, Andris Kleinhofs, Katrien M. Devos, Joachim Messing, Jeffrey L. Bennetzen

**Affiliations:** 1 Department of Biological Sciences Purdue University West Lafayette IN 47907 USA; 2 Purdue University Genomics Core S004 WSLR Purdue University West Lafayette IN 47907 USA; 3 Department of Agronomy & Range Science University of California Davis CA 95616 USA; 4 National Center for Genome Resources Santa Fe NM 87505 USA; 5 Department of Crop and Soil Sciences Washington State University Pullman WA 99164 USA; 6 PGIR-Waksman Institute Rutgers University Piscataway NJ 08855 USA; 7 John Innes Centre Norwich Research Park Colney Norwich NR4 7UH UK


**Comparative and Functional Genomics**
*Comp Funct Genom* 2002; **3**: 165–166.
Published online 21 March 2002 in Wiley InterScience (www.interscience.wiley.com). **DOI:** 10.1002/cfg.164

**Conference Review**

### Frequent genic rearrangements in two regions of grass genomes identified by comparative sequence analysis

Wusirika Ramakrishna[1]*, Jianxin Ma[1], Phillip SanMiguel[2], John Emberton[1], Jorge Dubcovsky[3], Bryan A Shiloff[4], Zeyu Jiang[4], Nils Rostoks[5], Carlos S Busso[3], Matthew Ogden[1], Eric Linton[6], Andris Kleinhofs[5], Katrien M Devos[7], Joachim Messing[6] and Jeffrey L Bennetzen[1]

[1] *Department of Biological Sciences, Purdue University, West Lafayette, IN 47907, USA*
[2] *Purdue University Genomics Core, S004 WSLR, Purdue University, West Lafayette, IN 47907, USA*
[3] *Department of Agronomy & Range Science, University of California, Davis, 95616 CA, USA*
[4] *National Center for Genome Resources, Santa Fe, NM 87505, USA*
[5] *Department of Crop and Soil Sciences, Washington State University, Pullman, WA 99164, USA*
[6] *PGIR-Waksman Institute, Rutgers University, Piscataway, NJ 08855, USA*
[7] *John Innes Centre, Norwich Research Park, Colney, Norwich NR4 7UH, UK*

**Correspondence to:*
*Department of Biological Sciences, Purdue University, West Lafayette, IN 47907, USA.*
*E-mail: wusirika@purdue.edu*

Grass genomes show extensive colinearity based on comparative genetic maps, although some large chromosomal rearrangements mark particular lineages. Small rearrangements involving one or a few genes would be missed by these comparative maps. Hence, we have undertaken sequence comparisons between BACs (bacterial artificial chromosomes) that contain 80–200 kb genomic segments of several grass species.

*Rp1* is a complex disease resistance locus in maize that provides race-specific resistance to the leaf rust disease caused by the fungus *Puccinia sorghi*. Sequence analysis of maize and sorghum *Rp1* BACs revealed two *Rp1* homologues and twelve other gene-homologous sequences, of which at least ten genes were truncated in one maize segment and eight gene-homologous segments were found in a second maize segment, of which two were *Rp1*-related and the other six were truncated. The truncated gene segments may have arisen by break repair, probably through homologous or illegitimate recombination. A 43 kb region with an *Rp1* homologue, six truncated genes and three *Opie* retrotransposons was duplicated on two maize BACs. Estimation of divergence times for the *Rp1* homologues, the three *Opie* elements and the intervening regions between duplicated regions are consistent with the duplication having occurred within the last 200 000 years. The Retrotransposons *Opie-B*, *Opie-C* and *Opie-D* were inserted before this duplication. In sorghum, the sequenced region includes a cluster of five *Rp1* homologues, of which two are truncated with N-terminal deletions. Of the other three *Rp1* homologues, a stop codon is present in one and a retrotransposon is inserted in another. The *Rp1*-homologous region in sorghum has several genes that are either duplicated, inverted, or both. Five duplicated genes other than the *Rp1* homologues are present. Physical mapping revealed the presence of eleven *Rp1* homologues that mapped to about 400 kb in B73 maize. Six *Rp1* homologues mapped to about 50 kb in sorghum inbred BT × 623 to a region on linkage group H that is colinear with maize chromosome 10.

The *Waxy1* gene encodes UDP-glucose starch glycosyl transferase, an enzyme that converts amylose to amylopectin. *Wx1* is in syntenic locations in all of these grass species, although the maize *Wx1* is within a paracentric inversion that places it near centromeric heterochromatin. Sequence analysis of

Copyright © 2002 John Wiley & Sons, Ltd.

BACs containing homologous *wx1* genomic regions in six grasses (barley, maize, pearl millet, rice, sorghum, and diploid wheat) revealed several rearrangements of gene content, order and/or orientation. Some of the rearrangements appear to mark specific lineages. A cluster of five genes that are 5′ to the *Wx1* loci are in the same relative order in the lineage that gave rise to maize, sorghum and pearl millet, but are in an inverted orientation in rice. None of the genes around the *Wx1* homologues in barley or wheat are homologues of any of the genes in the other four grass species studied.

Compared to other regions that we, and others, have studied, the *Wx1* and *Rp1* orthologous segments appear to be more highly rearranged. Studies of such rapidly evolving regions provide novel insights into the numerous mechanisms that create genomic diversity.

### Acknowledgement

This work was supported by the NSF Plant Genome Program (grants no. 9975618 and 9975793).

Copyright © 2002 John Wiley & Sons, Ltd. 

